# Impact Strength and Flexural Properties Enhancement of Methacrylate Silane Treated Oil Palm Mesocarp Fiber Reinforced Biodegradable Hybrid Composites

**DOI:** 10.1155/2014/213180

**Published:** 2014-08-31

**Authors:** Chern Chiet Eng, Nor Azowa Ibrahim, Norhazlin Zainuddin, Hidayah Ariffin, Wan Md. Zin Wan Yunus

**Affiliations:** ^1^Department of Chemistry, Faculty of Science, University Putra Malaysia, 43400 UPM Serdang, Selangor, Malaysia; ^2^Department of Bioprocess Technology, Faculty of Biotechnology and Biomolecular Sciences, University Putra Malaysia, 43400 UPM Serdang, Selangor, Malaysia; ^3^Chemistry Department, Centre for Defence Foundation Studies, National Defence University of Malaysia, Kem Sungai Besi, 57000 Kuala Lumpur, Malaysia

## Abstract

Natural fiber as reinforcement filler in polymer composites is an attractive approach due to being fully biodegradable and cheap. However, incompatibility between hydrophilic natural fiber and hydrophobic polymer matrix restricts the application. The current studies focus on the effects of incorporation of silane treated OPMF into polylactic acid (PLA)/polycaprolactone (PCL)/nanoclay/OPMF hybrid composites. The composites were prepared by melt blending technique and characterize the composites with Fourier transform infrared spectroscopy (FTIR), thermogravimetric analysis (TGA), and scanning electron microscopy (SEM). FTIR spectra indicated that peak shifting occurs when silane treated OPMF was incorporated into hybrid composites. Based on mechanical properties results, incorporation of silane treated OPMF enhances the mechanical properties of unmodified OPMF hybrid composites with the enhancement of flexural and impact strength being 17.60% and 48.43%, respectively, at 10% fiber loading. TGA thermogram shows that incorporation of silane treated OPMF did not show increment in thermal properties of hybrid composites. SEM micrographs revealed that silane treated OPMF hybrid composites show good fiber/matrix adhesion as fiber is still embedded in the matrix and no cavity is present on the surface. Water absorption test shows that addition of less hydrophilic silane treated OPMF successfully reduces the water uptake of hybrid composites.

## 1. Introduction

The oil palm (*Elaeis guineensis*) originates from South Africa which grows well in all tropical areas of the world and it has become one of the main industrial crops. Malaysian palm oil industry has grown tremendously over the last 25 years and Malaysia became the world's leading producer and exporter of palm oil [1]. Generally, oil palm biomass products are produced from two different sources which are from plantation and mills. Biomass form plantation is mainly truck and fronds while biomass from mill consists of empty fruit bunches (EFB), mesocarp fibers, palm kernel shell, and palm oil mill effluent (POME) [2]. For every kg of palm oil produced, approximately 4 kg of dry biomass is produced, excluding palm oil mill effluent (POME). In 2010, the amount of mesocarp fiber available was 10.80 Mt/year [3]. Therefore, there is huge amount of fiber that can be utilized instead of being discarded as waste. Traditionally, the mesocarp fiber is mixed with kernel shell and being utilized as solid fuel to generate electricity for the mill.

Recently, many studies have been conducted by academic or industrial researcher on biodegradable polymer in order to replace conventional nonbiodegradable polymer which causes major drawback to the environment. However, the cost of biodegradable polymer is comparatively higher than petrochemical based nonbiodegradable polymer that limits its application. The incorporation of cheap natural fiber as reinforcement filler into biodegradable polymer is an alternative to reduce its cost. Natural fiber as reinforcement filler in polymer composites has received increasing attention from researchers as natural fibers have many significant advantages over synthetic fibers. They are environmentally friendly, fully biodegradable, abundantly available, renewable, and cheap and have low density [4]. However, there are some disadvantages such as poor wettability, incompatibility with some polymeric matrices, and high moisture adsorption that restrict their usage in polymer composites [5].

In order to make hydrophilic natural fibers more compatible with hydrophobic polymer matrix, natural fiber has been modified to enhance the effectiveness of interfacial adhesion. Chemical modification of natural fiber exposes more reactive groups on the fiber surface and thus promotes more efficient coupling with polymer matrix [6]. Chemical modifications such as alkali treatment [7], acetylation treatment [8], isocyanate treatment [9], maleated coupling agents [10], silane coupling agents [11], and grafting [12] have been conducted to improve fiber matrix adhesion.

Silane coupling agents are a bifunctional molecule that are used to modify surface of natural fibers. The bifunctional silane molecules form chemical link between the matrix and the fibers by forming a chemical bond with the surface of fibers through a siloxane bridge while its organofunctional group bonds to the polymer matrix. When the silane bonded fiber surface connects with the matrix, the organofunctional groups on the fiber surface react with the functional groups that exist in the polymer matrix and form stable covalent bond with the matrix. Therefore, silane coupling agents function as a bridge between fibers and matrix [13]. Huda et al. modified kenaf fiber using alkalization and silane treatments and then blend with polylactic acid (PLA). It was found that both silane treated fiber reinforced composite and alkali treated fiber reinforced composite offered superior mechanical properties compared to untreated fiber reinforced composite [14].

Polylactic acid (PLA) is biodegradable polymer with good mechanical properties, thermal plasticity, and biocompatibility. However, PLA is a comparatively brittle and stiff polymer with low deformation at break. Therefore, modification of PLA is needed in order to compete with other flexible polymers such as polypropylene or polyethylene [15]. Polycaprolactone (PCL) is flexible semicrystalline biodegradable polymer with low melting point and exceptional blend-compatibility. High flexibility PCL can be considered as a good plasticizer for PLA compared to low molecular weight plasticizers as it does not migrate to the surface of the blended samples and the physical properties cannot be debased [16].

Hybrid composites are materials made by combining two or more different types of fillers in a single matrix. Although several fillers can be incorporated into the hybrid system, a combination of only two types of the fillers would be more beneficial. By careful selection of the reinforcing fillers/fibers, the performance properties of the resulting composite can be significantly improved while the material cost can be substantially reduced [17]. The properties of hybrid composites depend on the individual components in which there is a more favourable balance between the inherent advantages and disadvantages. Besides, the advantages of one type of filler could complement the other filler in hybrid composite that contains two or more types of fillers. Through proper material design, a hybrid composite with balance in cost and performance could be obtained [18].

In our preliminary studies [19, 20], we reported that the addition of 1 wt% hydrophilic nanoclay into PLA/PCL/OPMF biocomposites successfully improves tensile properties and flexural properties and impacts strength of biocomposites. FTIR spectra show that peak shifting is observed when 1 wt% of nanoclay was incorporated into biocomposites which indicate that there might be some physical interaction between PLA, PCL, clay, and OPMF in composites. TGA thermogram reveals that the addition of nanoclay improves the thermal stability of the biocomposites. Water sorption test shows that the addition of nanoclay improves water resistance of biocomposites.

In our previous paper [21], we successfully treated OPMF with methacrylate silane. FTIR spectra indicate that silane treated OPMF is less hydrophilic compared to unmodified OPMF. TGA thermogram shows that silane treated OPMF shows higher thermal stability than unmodified OPMF. SEM micrograph revealed that surface of silane treated OPMF are rough and porous while the surface of unmodified OPMF is smooth and sleek. We also reported that incorporation of silane treated OPMF in hybrid composites shows better tensile strength, tensile modulus, and elongation at break than unmodified OPMF hybrid composites [22].

The aim of incorporation of cheap natural fiber (OPMF) into expensive biodegradable polymer (PLA and PCL) was to reduce its cost. Due to the addition of hydrophilic fiber into hydrophobic matrix will reduce the mechanical strength of composites as incompatibility issue present between fiber and matrix; therefore objective of this paper is to investigate the effect of incorporation of methacrylate silane treated OPMF on mechanical, morphology, thermal, and water absorption properties of PLA/PCL/nanoclay/OPMF hybrid composites by melt intercalation. Various characterization techniques such as Fourier transform infrared spectroscopy (FTIR), thermogravimetric analysis (TGA), and scanning electron microscopy (SEM) were used to study the effect of incorporation of methacrylate silane treated OPMF on the properties of PLA/PCL/clay/OPMF hybrid composites.

## 2. Experimental

### 2.1. Materials

All reactions were carried out by using reagent grade chemicals (>98% purity) without further purification. The hydrophilic nanoclay (Nonamer PGV) was purchased from Sigma-Aldrich and used as received. Polylactide Resin 4060D was supplied by NatureWorks while Polycaprolactone (CAPA 650) was supplied by Solvay Caprolactone. Oil palm mesocarp fibers were obtained from Felda Palm Ind. Sdn Bhd., Serting Hilir, Negeri Sembilan, Malaysia. 3-(Trimethoxysilyl)propyl methacrylate was purchased from Acros Organic.

### 2.2. Processing of Raw OPMF

The raw OPMF fiber was soaked in distilled water for 24 h to remove impurities. It was then rinsed with hot water (60°C) twice and finally with acetone to remove wax prior to drying at 60°C in an air oven. The fiber was ground and sieved to a particle size of 150 *μ*m using a crusher machine.

### 2.3. Modification of OPMF by Methacrylate Silane

5 wt% of silane (weight percentage compared to the OPMF) was dissolved for hydrolysis in a mixture of 3 : 2 ratio of ethanol and water. The PH of the solution was adjusted to 4 with acetic acid and stirred continuously for 1 hour. The unbleached or bleached OPMF were soaked in the solution for 3 hours and then dried at 60°C in air oven overnight.

### 2.4. Preparation of Hybrid Composites

The composites were prepared by melt blending technique where the compositions of PLA and PCL were kept constant at 85 wt% and 15 wt%, respectively, in blend while the clay content constant was kept at 1 wt%. Only the content of OPMF (unmodified and silane treated) varies from 0% to 30%. The formulation table was shown in Table 1. PLA, PCL, nanoclay, and OPMF were manually premixed in a container and fed into Brabender Plastograph EC at 170°C with rotor speed of 50 rpm for 10 minutes. The products were then compression moulded into sheets of 1 mm (for tensile properties) or 3 mm (for flexural properties and Izod impact resistance) thickness by an electrically heated hydraulic press with a force of 1500 kN at 160°C for 10 minutes. The sample sheets were then used for further characterization.

### 2.5. Fourier Transform Infrared Spectroscopy (FTIR)

Perkin Elmer Spectrum 100 series spectrometer equipped with attenuated total reflectance (ATR) were used to determine the functional groups and types of the bonding of the samples with the infrared spectra were recorded in the range of frequency of 280 to 4000 cm^−1^ with the resolution of 4 cm^−1^ and the number of scans is 16 scans.

### 2.6. Tensile Properties

Tensile properties measurement was performed by Instron machine model 4301, with grip attachment distance of 45 mm. Load of 1.0 kN was applied at constant crosshead speed of 5 mm min^−1^. Computerized Instron (Software series 9, national instruments GPIB PC2/2a and NI-488.2) was used to process data. Test specimen was prepared and stamped in compliance with ASTM D638 dumbbell parameters. Sample thickness was measured with Mitutoyo Digimatic Indicator, type IDF-112, having measuring accuracy of ±0.001 mm.

### 2.7. Flexural Properties

The flexural strength and modulus were measured with Instron Universal Testing Machine 4301 according to ASTM D790. The size of the samples testing is 127 mm × 12.7 mm × 3 mm. The crosshead speed is 1.3 mm/min and the support span length is 48 mm. Data was processed with computerized Instron (Software series 9, national instruments GPIB PC2/2a and NI-488.2).

### 2.8. Izod Impact Strength

The Izod impact test was carried out according to ASTM D256 standard using an impact tester (IZOD Impact Tester). The sample size is 63.5 × 12.7 × 3 mm, while the notch length is 2.54 mm. The energy required to break the samples was divided by unit area of residual cross section of sample to obtain impact resistance value. The impact strength (J/m) was calculated by dividing the energy obtained (J) with the thickness of specimen (m).

### 2.9. Thermogravimetric Analysis (TGA)

TGA testing was conducted in accordance with ASTM E1131. Perkin Elmer TGA7 was used for thermogravimetric analysis of samples where about 15 mg of the samples were heated from 35°C to 800°C with the heating rate of 10°C/min. Nitrogen gas was pumped with the flow rate of 20 mL/min in order to let the analysis be carried out in nitrogen atmosphere.

### 2.10. Scanning Electron Microscopy (SEM)

The surface morphology of fracture surface was observed with SEM JEOL JSM-6400. The fracture surface was obtained from plain strain fracture tensile tested specimens and was sputter coated with gold using Bio-rad coating system before viewing.

### 2.11. Water Sorption Test

Water absorption studies were performed following the ASTM D638, Type M3 standard. The films were cut into dumbbell shape and dried at room temperature overnight to reach the constant weight. Then the samples were immersed at 25°C of distilled water for up to 30 days. Mass uptakes of the samples were measured periodically by removing them from the water bath. The samples were wiped with the tissue paper to remove the surface water. The moisture uptake expressed in percent weight gain, Δ*M*, is

(1) $$\begin{array}{c} \Delta M=\frac{M_{t}-M_{o}}{M_{o}}\times 100\%, \end{array}$$

where *M*_*t*_ is mass of sample after immersion and *M*_*o*_ is mass of sample before immersion.

## 3. Results and Discussion

### 3.1. Fourier Transform Infrared Spectroscopy (FTIR)

**Figure 1:**
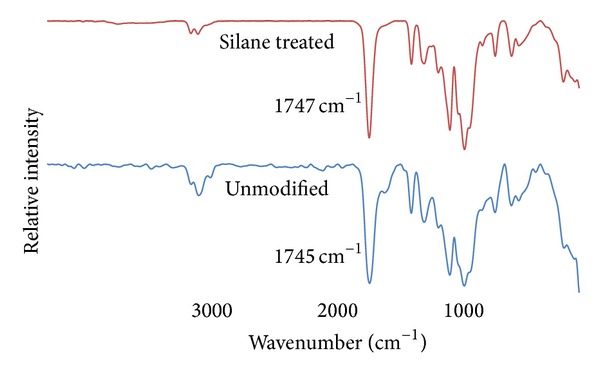
FTIR spectra of unmodified and silane treated OPMF hybrid composites.

FTIR spectra of different OPMF reinforced PLA/PCL/nanoclay/OPMF hybrid composites are illustrated in Figure 1. All hybrid composites exhibit strong absorbance peak around 1750 cm^−1^, respectively, indicated the presence of C=O stretching in composites spectra. C=O stretching peaks for unmodified and silane treated OPMF hybrid composites are 1745 cm^−1^ and 1747 cm^−1^, respectively. Results show that there is carbonyl stretching peak shifting in silane treated OPMF hybrid composites. Shifting of peaks in IR spectra is observed when there is interaction in polymer blends [23]. Therefore, this indicated that there are some physical interactions between methacrylate reactive groups of silane on surface of OPMF with polymer matrix.

### 3.2. Tensile Properties

In our previous studies, the effect of incorporation of silane treated OPMF on tensile properties of hybrid composite was investigated [22]. Tensile properties of unmodified and silane treated OPMF in PLA/PCL/nanoclay/OPMF hybrid composites are shown in Table 2. PLA85/PCL15 has better tensile properties compared to the hybrid composites as it is common that addition of hydrophilic fiber into hydrophobic matrix will reduce the tensile properties. However, the addition of 10% silane treated OPMF in composites (40.45 MPa) shows better tensile strength than unmodified OPMF (36.32 MPa) composites with the enhancement of about 11.37%. All composites show highest tensile strength at 10% fiber loading and then decrease when amount of fiber increases because the incorporation of fiber weakens the composites. Besides, weak bonding between hydrophilic filler with hydrophobic polymer matrix obstructs stress propagation of the composites [24]. The incorporation of silane treated OPMF shows better tensile strength than unmodified OPMF composites because the modified fibers are more hydrophobic than unmodified OPMF as proved in previous studies [21]. More hydrophobic fiber enhances compatibility between fiber and matrix, which increases strength, stiffness, and interfacial adhesion of composites [25].

Tensile modulus indicates the stiffness of materials. Incorporation of silane treated OPMF shows higher tensile modulus than unmodified OPMF because improvement in fiber/matrix adhesion provides better stress transfer in composites. All composites show highest tensile modulus at 10% of fiber loading which are 865.50 MPa and 685.80 MPa for silane treated and unmodified OPMF composites, respectively.

Silane treated OPMF composites show maximum elongation at break at 10% fiber loading with the elongation of 0.95 mm which is higher than unmodified OPMF composites (0.74 mm) and thus indicate that better fiber/matrix adhesion between silane treated OPMF with composites as enhance the tensile properties of composites is achieved. The further addition of fiber reduces the elongation at break because addition of fiber into matrix affects composites; they become stiffer and harder as the segment mobility of the composites is reduced [26].

### 3.3. Flexural Properties

**Figure 2:**
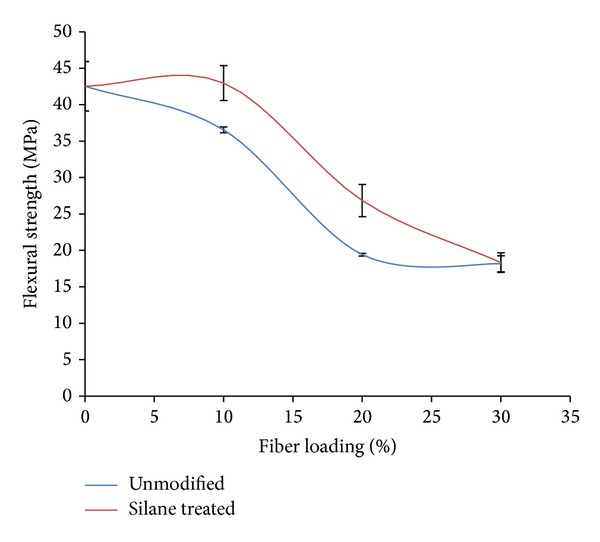
Flexural strength of unmodified and silane treated OPMF hybrid composites.

Figure 2 illustrated flexural strength of different OPMF in PLA/PCL/nanoclay/OPMF hybrid composites. The addition of 10% silane treated OPMF into composites enhances the flexural strength of unmodified OPMF composites from 36.53 MPa to 42.96 MPa with the enhancement of about 17.60%. This might be due to good adhesion between fiber and matrix as silane acts as bridge between fiber and matrix when fiber surface contact with matrix [27]. Flexural strength of silane treated OPMF composites shows higher value than tensile strength of composites, which might be due to the orientation of fibers in the outer layer of the composites [28]. Flexural strength decreases when higher amount of fiber is added into the composites due to the increment of population of fiber defects and fiber ends with increased fiber content [29].

**Figure 3:**
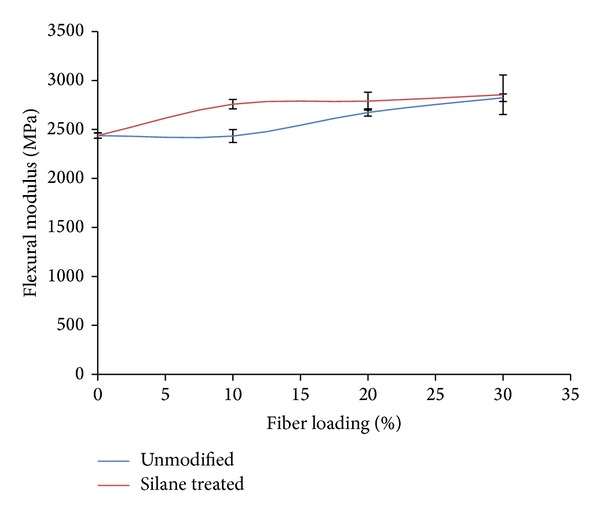
Flexural modulus of unmodified and silane treated OPMF hybrid composites.

Flexural modulus of different OPMF in PLA/PCL/nanoclay/OPMF hybrid composites is shown in Figure 3. Flexural modulus of composites increases from 2437 MPa to 2758 MPa when 10% of silane treated OPMF is added into composites. The silane treated OPMF composites show increment of flexural modulus at higher fiber loading which agree with the findings of Sawpan et al. [29]. Due to more compatibility between less hydrophilic fiber with matrix that present in the continuous interfacial, better and efficient stress transfer in composites is achieved and thus silane treated OPMF increases the flexural modulus of composites [25].

### 3.4. Izod Impact Strength

**Figure 4:**
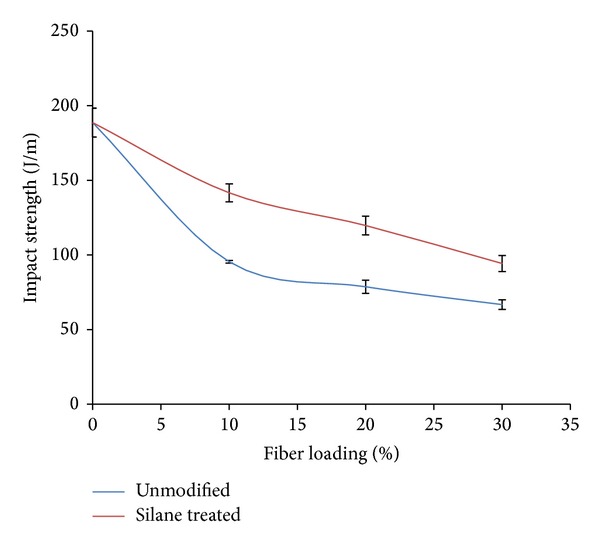
Impact strength of unmodified and silane treated OPMF hybrid composites.

Impact strength of modified OPMF in PLA/PCL/nanoclay/OPMF hybrid composites is illustrated in Figure 4. All composites show highest impact strength at 10% fiber loading while silane treated OPMF shows higher impact strength (141.66 J/m) than unmodified OPMF (95.44 J/m) with the improvement of around 48.43%. Incorporation of fiber into matrix results in reduction in impact strength as fiber tends to hinder deformation and ductile mobility of polymer molecules which reduce the capability of composites to absorb energy during crack propagation [30]. Silane treated OPMF improve fiber wettability resulting in less void (crack initiation site) present in composites. Therefore, fewer flaws in composites due to less void spaces result in improvement in impact strength of composites [25].

### 3.5. Thermogravimetric Analysis (TGA)

**Figure 5:**
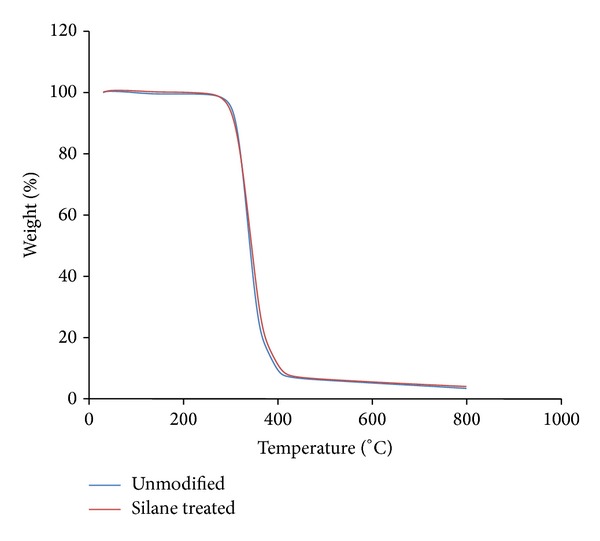
TG thermogram of unmodified and silane treated OPMF hybrid composites.

**Figure 6:**
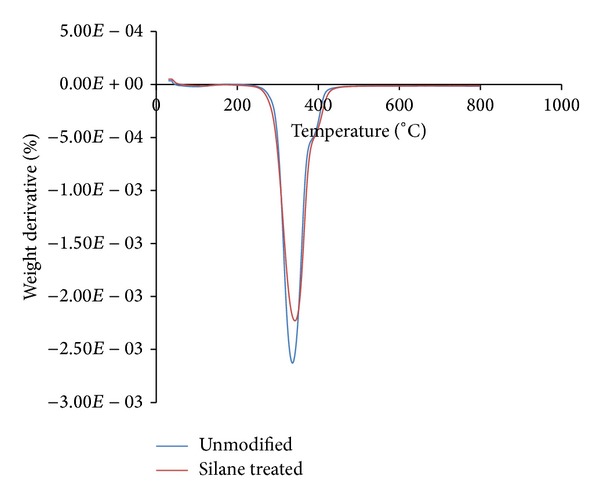
DTG thermogram of unmodified and silane treated OPMF hybrid composites.

Figures 5 and 6 show the TG and DTG thermogram of different OPMF in PLA/PCL/nanoclay/OPMF hybrid composites. The result shows that silane treated OPMF has no effect in improvement of thermal stability of composites with the onset temperature at 217.67°C. Although our previous studies [21] show that the silane treated OPMF shows higher thermal stability than unmodified OPMF, the incorporation of silane treated OPMF did not show increment in thermal stabilities. This might be because the amount of fiber incorporation is low (30%) in composites, which therefore is not a very influencing factor to improve the thermal stability of hybrid composites.

### 3.6. Scanning Electron Microscopy (SEM)

**Figure 7:**
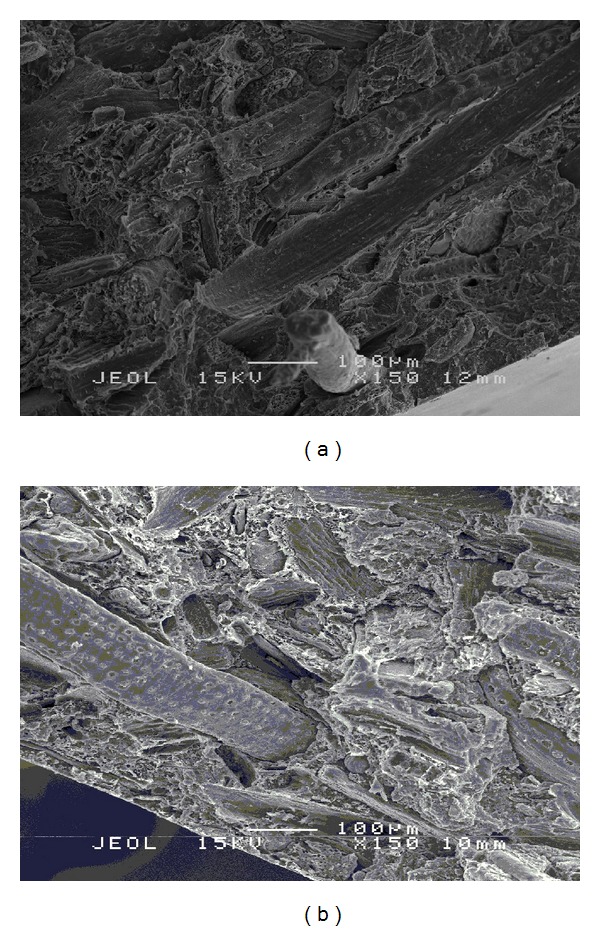
SEM micrograph of (a) unmodified and (b) silane treated OPMF hybrid composites.

SEM micrograph of fractured surface of (a) unmodified and (b) silane treated OPMF hybrid composites is shown in Figure 7 at magnification of 150X.  Figure 7(a) shows that unmodified OPMF hybrid composites show good fiber/matrix adhesion as no cavity present and fiber breakage could be seen on facture surfaces.  Figure 7(b) indicates that silane treated OPMF hybrid composite also shows that good adhesion exists at interphase as silane treated fiber is still embedded in the matrix. Besides, no cavity present on the surface indicates that no fiber has been pulled out during tensile test which indicates good fiber/matrix adhesion.

### 3.7. Water Sorption Test

**Figure 8:**
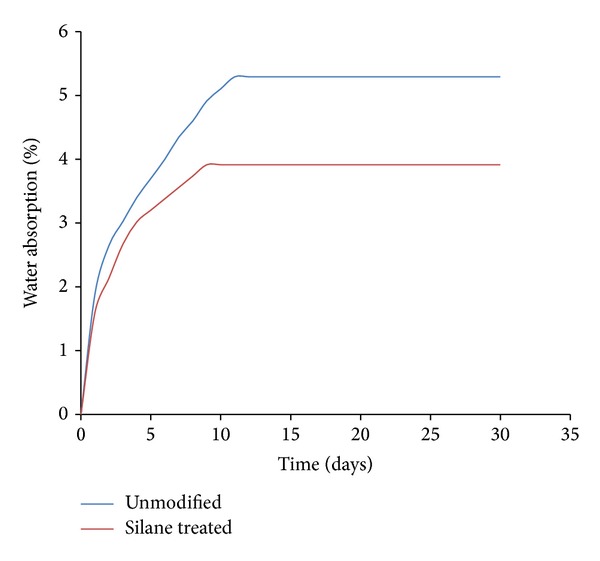
Water absorption of unmodified and silane treated OPMF hybrid composites.

Water absorption of different OPMF in PLA/PCL/nanoclay/OPMF hybrid composites is shown in Figure 8. The fiber absorbs water due to the presence of hydroxyl groups which absorb water through the formation of hydrogen bonding [31]. All composites show a sharp increment in water absorption at the beginning and then remain constant around 10 days with the maximum water absorption being 5.29% and 3.91% for unmodified and silane treated OPMF hybrid composites, respectively. Water absorption of all composites is typical type of Fickian diffusion behaviour. In Fick's law, the concentration gradient is the driving force for diffusion and amount of the component diffused in a function of time. Generally, moisture absorption processes follow the prediction of Fick's law as the mass of water absorbed increases linearly with square root of time and then gradually shows until equilibrium plateau [32]. Silane treated OPMF shows lower water absorption rate because modified fiber is less hydrophilic as numbers of hydrophilic hydroxyl groups reduce by react which silane which leads to exclusion of water from substrate [31].

## 4. Conclusion

The incorporation of methacrylate silane treated OPMF successfully enhances the impact and flexural properties of PLA/PCL/nanoclay/OPMF hybrid composites. FTIR spectra indicated that peak shifting occurs when silane treated OPMF was incorporated into hybrid composites which indicates that there are some interactions between methacrylate reactive groups of silane on surface of OPMF with polymer matrix. Incorporation of silane treated OPMF enhances the flexural and impact strength of unmodified OPMF hybrid composites around 17.60% and 48.43%, respectively, at 10% fiber loading as the incorporation of more hydrophobic silane treated fiber improves the fiber/matrix interaction with hydrophobic polymer matrix. TGA thermogram shows that incorporation of silane treated OPMF did not show increment in thermal properties of hybrid composites. SEM micrographs revealed that silane treated OPMF still embedded in the hybrid composites and no cavity present on the surface shows that no fiber been pulled out during tensile test which indicate good fiber/matrix adhesion between silane treated OPMF with matrix. Water absorption test shows that addition of silane treated OPMF successfully reduces the water uptake of hybrid composites as silane treated OPMF is more hydrophobic. Therefore, the incorporation of silane treated OPMF in hybrid composites can enhance the mechanical properties of composites while in the same time the cheap silane treated OPMF can lower the cost of biodegradable polymer which widens the application of biodegradable polymer based composites as it is comparable to conventional nonbiodegradable polymer. The applications of the hybrid composites might be more suitable for single-use materials that do not require very high strength, while at the same time biodegradability and being environment friendly are one of the concerns of selection of materials.

## Figures and Tables

**Table 1:** PLA/PCL/clay/OPMF composition.

Samples	PLA (g)	PCL (g)	Clay (g)	OPMF (g)
PLA/PCL/1 wt% clay/10 wt% OPMF	22.72	4.01	0.27	3.00
PLA/PCL/1 wt% clay/20 wt% OPMF	20.20	3.56	0.24	6.00
PLA/PCL/1 wt% clay/30 wt% OPMF	17.67	3.1	0.21	9.00

**Table 2:** Tensile properties of unmodified and silane treated OPMF hybrid composites.

Sample	Tensile strength (MPa)	Elongation at break (mm)	Tensile modulus (MPa)
PLA85/PCL15	45.94 ± 1.75	1.68 ± 0.30	902.92 ± 50.69
Unmodified OPMF hybrid composites			
PLA85/PCL15/1 wt% clay/10 wt% OPMF	36.32 ± 1.22	0.74 ± 0.02	685.80 ± 71.32
PLA85/PCL15/1 wt% clay/20 wt% OPMF	32.32 ± 0.55	0.64 ± 0.10	661.17 ± 46.36
PLA85/PCL15/1 wt% clay/30 wt% OPMF	29.46 ± 1.58	0.61 ± 0.02	577.18 ± 27.04
Silane treated OPMF hybrid composites			
PLA85/PCL15/1 wt% clay/10 wt% OPMF	40.45 ± 0.94	0.95 ± 0.01	865.50 ± 50.24
PLA85/PCL15/1 wt% clay/20 wt% OPMF	33.18 ± 0.86	0.63 ± 0.06	842.55 ± 45.63
PLA85/PCL15/1 wt% clay/30 wt% OPMF	29.35 ± 0.53	0.62 ± 0.02	767.70 ± 56.50
